# Trauma as Pathogenesis of a Plantar Forefoot Fibrolipoma: First Case and Review of the Literature

**DOI:** 10.1155/2013/691276

**Published:** 2013-12-24

**Authors:** Juliana S. P. Vasconcelos, Faris Gharaibeh, Hans-Christoph Nittinger

**Affiliations:** Department of Surgery, Agaplesion Ev. Bathildis Hospital, 31812 Bad Pyrmont, Germany

## Abstract

Lipomas and their variants are benign soft-tissue tumors that occur at any age and most frequently on the upper back and neck, shoulder, and abdomen. The foot is a relatively uncommon site for soft-tissue neoplasia and the etiology is not usually related to trauma. We describe a case of a pedunculated fibrolipoma of the forefoot that originated from a cut wound at the Atlantic Ocean. A brief review of the literature is also given.

## 1. Case Report

**Figure 1:**
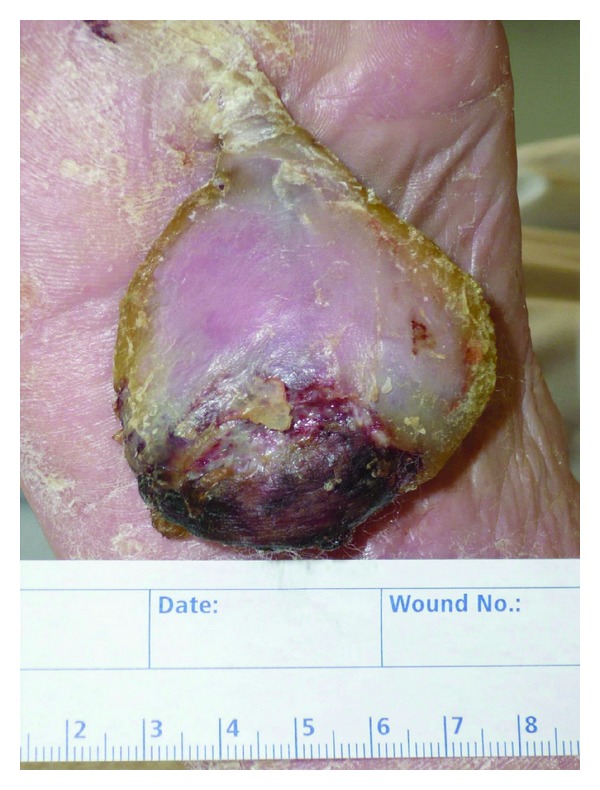
Ulcerated fibrolipoma of the plantar surface.

A 92-year-old Caucasian woman presented a slowly increasing pedunculated soft-tissue tumor at the forefoot sole of her right foot. The lesion had approximately 8,5 cm length, 5 cm width, and 2-3 cm thickness and demonstrated an ulcerated surface (Figure 1). The original lesion was acquired 4 years before at the Fuerteventura Island. After swimming in the Atlantic Ocean, the patient noticed a little flap hanging on her foot sole without active bleeding. Since then, the flap has been growing slowly and spontaneously. Once she did not present any pain or functional disturbance, she did not get to the doctor.

After four years, she felt local pain when walking and then went to the hospital. The patient had no diagnosed comorbidities. There were no vascular or neurological symptoms. X-ray of the foot did not reveal any bony erosion adjacent to the tumor.

**Figure 2:**
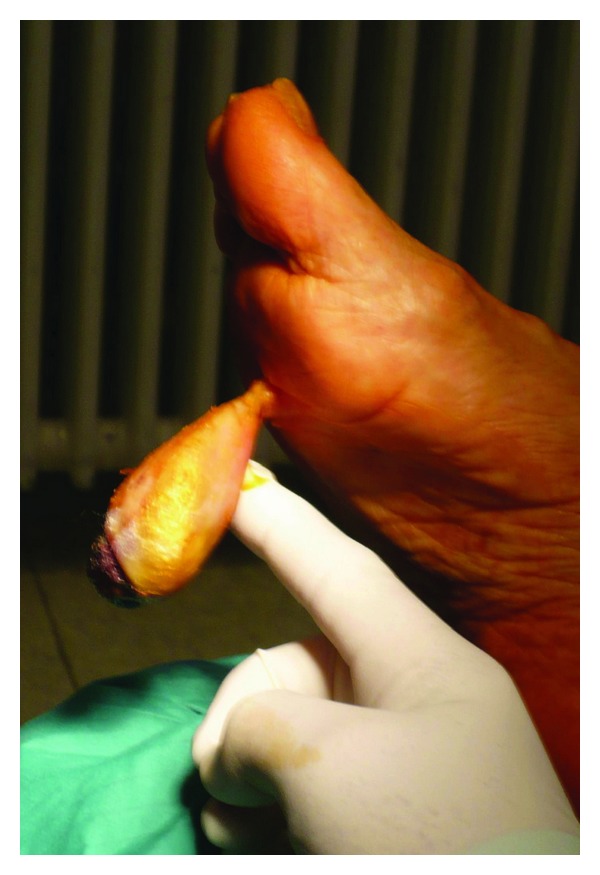
Rare pedunculated tumor of the plantar surface.

Surgical excision on the shaft basis was performed with local anaesthesia and sedation (Figure 2). The wound was primarily closed. The postoperative course showed no complications. The patient's mobility was restored on the second postoperative day with the help of a wound care shoe. At the six-month followup there was no recurrence and the patient was asymptomatic.

The histological analysis was confirmed from two different pathology institutes, the second and last one being a reference centre for soft-tissue tumors in Germany. They described an ulcerated fibrolipoma of the plantar surface of the foot.

## 2. Discussion

Lipomas comprise a relatively uncommon tumor of the foot, despite being one of the most common soft tissue tumors in other areas of the body. Lipomas can occur at any age, although the fifth and sixth decades of life are the most affected. Statistics as to sex incidence vary and a higher incidence of lipomas and their variants in one gender could not yet be confirmed [1–3].

Lipomas and its variants represent approximately 8–16% of all benign soft-tissue tumors and around 3-4% of all tumors that arise in the foot [1, 3, 4]. Kirby et al. found 67% of foot lipomas to occur in the ankle region and 33% on the dorsum of the foot [4]. Another author also found the majority (33%) to occur around the ankle or heel. Only 9% present in the hallux, digits, sole, or dorsal surface of the foot [5].

Lipomas have to be differentiated from malign soft-tissue tumors of the foot such as Kaposi's sarcoma, synovial sarcoma, or liposarcoma. These tumors may be associated with metastasis and a considerable disease-related mortality rate [6].

Patients with an indeterminate lesion should be evaluated with radiography, magnetic resonance imaging or may be submitted to a biopsy of the mass. The ultimate goal of treatment is the tumor elimination and restoration of the patient's long-term mobility and function [7]. In our case, the pedunculated tumor showed no clinical or radiographic signs of malignancy and the excisional biopsy was performed for the definitive histological diagnosis and treatment of the patient. The mobility was completely restored after surgery.

The exact etiology of lipomas and their variants is not well established in the literature. An association with genetic aberrations has been described for most types of adipocytic tumor [8, 9] and some trauma mechanisms, such as blunt trauma or chronic intermittent compression, have been associated with the apparition of subcutaneous or gastrointestinal lipomas [10–14].

The etiopathogenetic mechanism of posttraumatic lipomas is still unknown. The most accredited mechanism is the herniation or relocation of normal deeper fat through Scarpa's layer secondary to trauma, as first suggested by Brooke and MacGregor [15]. They called this mass “pseudolipoma,” which differs from a lipoma in that it does not have a capsule.

Rozner and Isaacs suggested that an etiologic mechanism could be a scar contracture after a shearing fascial injury [16]. Finally, Signorini and Campiglio cited the differentiation of mesenchymal precursors (preadipocytes) to mature adipocytes by trauma [10] and proposed this as a possible etiology of posttraumatic lipomas. Many factors such as local or systemic growth factors, inflammatory mediators, or products of degradation of hematoma or fat necrosis could trigger this differentiation [10, 17].

In our case, more than just one etiologic mechanism may be involved. It is reasonable to suppose that the primary wound flap due to the cut trauma might have led to the formation of a scar tissue. Further, this tissue suffered some years of chronic compression due to its location at the plantar surface, what may have led to an increasing local inflammatory process. All these factors could have finally induced the proliferation and differentiation of preadipocytes into mature cells and the expansion of the mass.

To our knowledge, however, the implication of a cut trauma in the pathogenesis of plantar pedunculated fibrolipomas has not yet been described in the international literature. The real etiologic mechanism of posttraumatic lipomas remains uncertain.

In conclusion, the plantar tumors of soft tissues are a rare condition and are characterized by a slow and asymptomatic evolution, which delays their diagnosis. Although rare, lipomas and their variants should be considered in the differential diagnosis of tumors of the forefoot.

To our best knowledge, this is the first case of the implication of trauma in the pathogenesis of a plantar fibrolipoma ever reported in the international literature.

## References

[1] Bakotic BW, Borkowski P (2001). Primary soft-tissue neoplasms of the foot: the clinicopathologic features of 401 cases. *Journal of Foot and Ankle Surgery*.

[2] Enzinger FM, Enzinger FM, Weiss SW (1995). Benign lipomatous tumours. *Soft Tissue Tumours*.

[3] Kransdorf MJ (1995). Benign soft-tissue tumors in a large referral population: distribution of specific diagnoses by age, sex, and location. *American Journal of Roentgenology*.

[4] Kirby EJ, Shereff MJ, Lewis MM (1989). Soft-tissue tumors and tumor-like lesions of the foot. An analysis of eighty-three cases. *Journal of Bone and Joint Surgery A*.

[5] Berlin SJ (1984). A laboratory review of 67,000 foot tumors and lesions. *Journal of the American Podiatry Association*.

[6] Zeytoonjian T, Mankin HJ, Gebhardt MC, Hornicek FJ (2004). Distal lower extremity sarcomas: frequency of occurrence and patient survival rate. *Foot and Ankle International*.

[7] DeGroot H (2008). Approach to the management of soft tissue tumors of the foot and ankle. *Foot & Ankle Specialist*.

[8] Bridge JA, Cushman-Vokoun AM (2011). Molecular diagnostics of soft tissue tumors. *Archives of Pathology and Laboratory Medicine*.

[9] Hameed M (2007). Pathology and genetics of adipocytic tumors. *Cytogenetic and Genome Research*.

[10] Signorini M, Campiglio GL (1998). Posttraumatic lipomas: where do they really come from?. *Plastic and Reconstructive Surgery*.

[11] Zschiedrich M, Neuhaus P (1990). Pedunculated giant lipoma of the esophagus. *American Journal of Gastroenterology*.

[12] Coban YK, Uzel M, Gumus N (2006). Lipoma due to chronic intermittent compression as an occupational disease. *Annals of Plastic Surgery*.

[13] Copcu E (2004). Posttraumatic fingertip lipoma. *Plastic and Reconstructive Surgery*.

[14] Aust MC, Spies M, Kall S (2007). Lipomas after blunt soft tissue trauma: are they real? Analysis of 31 cases. *British Journal of Dermatology*.

[15] Brooke RI, MacGregor AJ (1969). Traumatic pseudolipoma of the buccal mucosa. *Oral Surgery, Oral Medicine, Oral Pathology*.

[16] Rozner L, Isaacs GW (1977). The traumatic pseudolipoma. *Australian and New Zealand Journal of Surgery*.

[17] Copcu E, Sivrioglu NS (2003). Posttraumatic lipoma: analysis of 10 cases and explanation of possible mechanisms. *Dermatologic Surgery*.

